# Effects of intramuscular and vaginal progesterone supplementation on frozen-thawed embryo transfer

**DOI:** 10.1038/s41598-019-51717-5

**Published:** 2019-10-24

**Authors:** Lei Jiang, Zhuo-Ye Luo, Gui-Min Hao, Bu-Lang Gao

**Affiliations:** The Second Hospital, Hebei Medical University, 215 West Heping Road, Shijiazhuang, 050000 Hebei Province P.R. China

**Keywords:** Infertility, Outcomes research

## Abstract

This study was to investigate effects of progesterone vaginal sustained-release gel and intramuscular injection of progesterone on frozen-thawed embryos transfer in 3013 patients receiving vaginal progesterone sustained-release gel and progesterone injection in artificial cycle for frozen-thawed embryo transfer. All patients were divided into two groups: group A with progesterone intramuscular injection (60 mg/d) plus dydrogesterone (10 mg tid) and group B with progesterone vaginal sustained-release gel of progesterone (90 mg/d) plus dydrogesterone (10 mg tid). There were 1988 women in group A treated with progesterone injection and 1025 women in group B with progesterone vaginal sustained-release gel. There were no statistically (*P* > 0.05) significant difference between the two groups in age, years of infertility, body mass index, endometrial thickness at transfer time, the average numbers of embryo transferred, cause of infertility, number of cycles, pregnancy rate and ectopic pregnancy rate. No significant (P > 0.05) differences existed in the clinical pregnancy (52.5% vs. 56.0%) and ectopic pregnancy (2.2% vs. 3.0%) rate between groups A and B. However, group B with vaginal progesterone supplementation had significantly (P < 0.05) greater implantation (37.0% vs 34.4%), delivery (45.1% vs. 41.0%) and live birth (45.0% vs. 40.8%) rate than group A with intramuscular progesterone injection, whereas group A had significantly (P < 0.05) greater early abortion rate (19.4% vs. 15.3%) than group B. This study showed that vaginal gel progesterone supplementation has good effects on frozen-thawed embryo transfer and can significantly increase the rate of implantation, delivery and live birth but decrease the abortion rate compared with intramuscular progesterone injection.

## Introduction

Trounson *et al*.^1^ were the first to report a live birth following frozen-thawed embryo transfer (FET) in 1983, and ever since then, embryo preservation and FET procedures have been increasingly applied for the treatment of infertility^2–8^, increasing the cumulative pregnancy rate but reducing the risk of hyper-stimulation syndrome of ovary and repeated pregnancies. Moreover, the FET procedure also provides a chance for women with genetic diseases or premature ovarian failure^9^. Currently, the FET procedure accounts for 25% of births with use of assisted reproductive techniques^8^, and embryos with high-quality are the crucial factor in increasing the chance of live birth in FET cycles besides good coordination between the transferred embryos and the endometrium in the pre-implantation cycle^10^. Progesterone is very important in creating harmony between the embryo and the endometerium and can reduce the abortion rate in the initial stage of pregnancy^8^. Progesterone at the luteal stage can ameliorate the outcome of fertility and has been used as the standard treatment in artificial reproductive techniques. Because the corpus luteum cannot be formed endogenously in the FET cycles owing to absence of ovulation, endometrial transformation of secretion before transfer of embryo and development of normal embryos after transfer depend on external supply of progesterone^7^. Nowadays, there are three approaches for progesterone support in the luteal phase: intramuscular injection, oral and vaginal supplementation. Oral supplementation is convenient but may not achieve secretory transformation of the endometrium because it has low bioavailability caused by hepatic first-pass effect^11^. Intramuscular injection of progesterone is often used in FET cycles to achieve both a high serum level and a high clinical pregnancy rate despite its inconvenience, local pain and inflammation at the injection site^12^. Some medical centers apply vaginal progesterone supplementation for luteal phase support since it has a high concentration of progesterone locally to exert a direct effect on the endometrium even though some limitations also exist including vaginal irritation, discharge and multiple applications during the day^8^. Currently, controversy exists with regard to the approach of progesterone supplementation and its clinical effects on clinical pregnancy, implantation, early abortion, delivery and live birth rates. Because few studies in the literature have been performed to investigate the best approach for progesterone supplementation, this study was to serve this goal by reviewing a large volume of patients who had vaginal supplementation of progesterone for artificial cycle progesterone replacement for FET assisted pregnancy therapy in our hospital. Since the combination of two luteal support drugs can reduce side effects and absorption problems caused by large doses of one single drug and can also reduce the rate of early vaginal bleeding after transplantation, dydrogesterone (10 mg tid, Dphaston, Abbott Healthcare, USA) was added in the study.

## Methods

This study was approved by the ethics committee of the Second Hospital of Hebei Medical University with all patients given their signed informed consent for FET assisted pregnancy therapy and participating in the study. All methods were performed in accordance with the relevant guidelines and regulations. Inclusion criteria were women between 20 and 40 years of age who received FET assisted pregnancy therapy in our hospital from January 2012 to August 2017, with an endometrial thickness ≥7 mm on the secretory transformation day and with progesterone supplementation for luteal phase support through either intramuscular injection of progesterone (60 mg/d) plus oral dydrogesterone (10 mg tid, Dphaston, Abbott Healthcare, USA) or vaginal progesterone sustained-release gel (90 mg/d) plus dydrogesterone (10 mg tid). Exclusion criteria were women with uterine diseases, submucous myoma, a history of spontaneous abortions or embryo transfer failure on over three occasions, medication affecting reproductive or metabolic functions and an endometrial thickness <7 mm on the secretory transformation day.

Based on the progesterone administration approach, patients were divided into group A with progesterone intramuscular injection (60 mg/d) plus dydrogesterone (10 mg tid) and group B with progesterone vaginal sustained-release gel of progesterone (90 mg/d) plus dydrogesterone (10 mg tid). In group A, intramuscular injection of progesterone (60 mg/d) plus dydrogesterone (10 mg tid) was administered daily from FET to 45 days after transfern. In group B, the progesterone vaginal sustain-release gel (90 mg/d) plus dydrogesterone (10 mg tid) was applied daily from FET to 45 days following transfer. In these two groups, the fresh cycle was treated with three protocols of controlled ovarian stimulation including agonist long, long-acting gonadotropin-releasing hormone agonist (GnRH-a) and antagonist protocols. Triptorelin was used as the agonist long protocol in the dose of 0.1 mg/d for 14 to 18 days in the mid-luteal phase. Leuproelin was used for the long-acting GnRH-a protocol in the dose of 3.75 mg starting from the second day of menstruation. Cetrorelix was used for the antagonist protocol in the dose of 0.25 mg from the sixth day of Gn to hCG day.

Patients receiving estrogen and progestogen replacement were treated with estradiol valproate (Progynova, Bayer, Germany) 2 mg–4 mg bid for 10–15 days since the third day of menstruation, and then, progesterone intramuscular injection plus dydrogesterone or progesterone vaginal sustained-release gel plus dydrogesterone was administered for transforming the endometrium. Either D3 embryos were transplanted four days following endometrium transformation or D5 blastocyts were transplanted six days after endometrium transformation. One to three embryos were transplanted each time on the transfer day. Two weeks following transfer, venous blood was drawn for test of beta-hCG. If the beta-hCG was ≥30 miu/ml, biochemical pregnancy was confirmed. Twenty to thirty days after transfer, transvaginal B-type ultrasonography was performed for confirmation of clinical pregnancy (gestational sacs) or ectopic pregnancy. Follow-up was conducted one year later about any delivery and infant health status.

### Statistical analysis

The SPSS 19.0 software (IBM, Chicago, IL, USA) was used for statistical analysis. Data were expressed as mean ± standard deviation (SD) or percentage (%). The measurement data were analyzed by t test, and the counting data were analyzed by χ^2^ test. P < 0.05 was considered statistically significant.

## Results

A total of 3013 patients who received FET assisted pregnancy therapy were retrieved including 1988 women in group A treated with progesterone injection and 1025 women in group B with progesterone vaginal sustained-release gel. The two groups were matched with no significant (P > 0.05) differences in age (30.22 ± 4.45 vs. 29.96 ± 4.53 years), infertility duration (3.79 ± 2.84 vs. 3.71 ± 2.79 years), body mass index (BMI, 23.26 ± 3.72 vs. 23.22 ± 3.76 kg/m^2^), endometrial thickness (9.67 ± 1.42 vs. 9.62 ± 1.56 mm) on transfer day, number of cycles (1.41 ± 0.73 vs. 1.40 ± 0.74) and causes of infertility (Table 1). There was no significant difference (P = 0.313) in the proportion of patients in different fresh cycles between the two groups.

**Table 1:** Demography of patients.

Group	A	B	P
Cases (%)	1988 (65.98%)	1025 (34.02%)	—
Age (year)	30.22 ± 4.45	29.96 ± 4.53	0.131
Infertility duration (year)	3.79 ± 2.84	3.71 ± 2.79	0.461
BMI (kg/m²)	23.26 ± 3.72	23.22 ± 3.76	0.781
Endometrial thickness on transfer day (mm)	9.67 ± 1.42	9.62 ± 1.56	0.376
Number of cycles	1.41 ± 0.73	1.40 ± 0.74	0.723
Number of embryos transferred	1.92 ± 0.31	1.94 ± 1.35	0.417
Type of infertility			0.15
Primary (%)	774 (38.9%)	370 (36.1%)	0.129
Secondary (%)	1214 (61.1%)	655 (63.9%)	
Pelvic and tubal factors	1085 (54.6%)	534 (52.1%)	
Ovulation obstacle	169 (8.5%)	85 (9.3%)	
Impaired ovarian reserve	24 (1.2%)	22 (2.1%)	

Note: BMI, body mass index. Group A had progesterone intramuscular injection (60 mg/d) plus dydrogesterone (10 mg tid) while group B had progesterone vaginal sustained-release gel of progesterone (90 mg/d) plus dydrogesterone (10 mg tid).

No significant (P > 0.05) differences existed in the clinical pregnancy (52.5% vs. 56.0%) and ectopic pregnancy (2.2% vs. 3.0%) rate between groups A and B (Table 2). However, group B with vaginal progesterone supplementation had significantly (P < 0.05) greater implantation (37.0% vs 34.4%), delivery (45.1% vs. 41.0%) and live birth (45.0% vs. 40.8%) rate than group A with intramuscular progesterone injection, whereas group A had significantly (P < 0.05) greater early abortion rate (19.4% vs. 15.3%) than group B.

**Table 2:** Pregnancy outcomes in both groups.

Group	A	B	P
Clinical pregnancy rate (%)	1044/1988 (52.5%)	574/1025 (56.0%)	0.069
Implantation rate (%)	1400/4071 (34.4%)	787/2126 (37.0%)	0.040
Early abortion rate (%)	203/1044 (19.4%)	88/574 (15.3%)	0.039
Ectopic pregnancy rate (%)	23/1044 (2.2%)	17/574 (3.0%)	0.641
Delivery rate (%)	815/1988 (41.0%)	462/1025 (45.1%)	0.032
Live birth rate (%)	811/1988 (40.8%)	461/1025 (45.0%)	0.028

Note: Group A had progesterone intramuscular injection (60 mg/d) plus dydrogesterone (10 mg tid) while group B had progesterone vaginal sustained-release gel of progesterone (90 mg/d) plus dydrogesterone (10 mg tid).

## Discussion

In artificial reproductive technologies, the implantation, pregnancy and live birth rates have remained quite low and have been affected by a lot of factors including women’s age, endometrial receptivity, progesterone treatment duration before embryo transfer, embryonic quality and development stage at transfer, cryopreservation method and ovarian stimulation with gonadotropins upon endometrium^13–18^. In this study, we investigated the effects of two approaches for progesterone supplementation for luteal phase support in FET and found that vaginal progesterone supplementation could significantly (P < 0.05) increase the implantation, delivery and live birth rate while decreasing early abortion rate compared with intramuscular progesterone injection in a cohort of Chinese women of around 30 years of age. The age of this infertile cohort seems younger than in other studies, however, this is the real situation in China. According to a survey in China on 1262 urban women in four cities of China, the average age of ideal first marriage for urban women is 24.77 years and the average interval between the ideal first marriage and the first birth is 1.09 years^19^. This situation makes the infertile women receiving FET younger in China than in other countries.

After investigating four approaches of support at the luteal phase consisting of vaginal progesterone, oral administration of dydrogesterone, combined supplementation of oral dedrogesterone and GnRH-analog, and joint application of oral dydrogesteone and human chorionic gonadotrophin, Zarei *et al*. found that vaginal progesterone is an acceptable regimen for luteal phase support like dydrogesterone plus GnRH-analog or human chorionic Gn in improving the clinical pregnancy rate compared with oral dydrogesterone alone^8^. In a global network-based survey to compare the clinic practice in luteal phase support in a total number of 51,155 cycles of *in vitro* fertilization per year in 35 countries, vaginal supplementation of progesterone has been used alone in 64% of the cycles or together with either oral supplementation or intramuscular injection of progesterone in 16% of the cycles for support at the luteal phase, indicating wide acceptance of vaginal progesterone supplementation^20^. Another prospective study showed that vaginal supplementation of progesterone for luteal phase support had significantly greater rates of pregnancy (70.9% versus 64.2%) or delivery (51.7% versus 45.4%) than intramuscular injection of progesterone^21^. Wang *et al*. studied the effect of vaginal progesterone gel in contrast to intramuscular injection of progesterone for support at the luteal phase and found that similar pregnancy outcomes had been achieved using both methods, suggesting that vaginal progesterone gel supplementation is a practical alternative approach to intramuscular injection^7^. High-dose vaginal progesterone supplementation has a better effect in increasing the rate of clinical pregnancy or live birth. This has been proved by the study by Enatsu *et al*. who found that a high dose of 1200 mg/d of vaginal progesterone had led to a significantly greater rate of clinical pregnancy of 63.2% and a higher live birth rate of 40.4% compared to 57.5% and 34.8%, respectively, in a lower dose of 900 mg/d^2^.

Supplementation of progesterone has developed into the standard therapy for support at the luteal phase in artificial reproductive technologies to enable transformation of endometrial mucosa and to promote embryonic survival via an effect of anti-inflammation mediated by nitric oxide synthesis^3,22^. Many medications have been available for luteal phase support^2^. Traditionally, intramuscular injection of progesterone was the most extensively applied route for support at the luteal phase due to reported evidence of increased rates of clinical pregnancy and delivery compared with those via vaginal routs^23^. Nonetheless, with accumulated evidence of the vaginal supplementation route as equally effective as the intramuscular one, vaginal progesterone application became a more popular and mostly widely used method in most countries^24,25^. Moreover, besides the inconvenience in use of injection, local pain and inflammation at the injection site, large amounts of progesterone through intramuscular injection are metabolized through the liver, resulting in unstable serum drug concentration and decreased bioavailability^12^. The vaginal route of progesterone supplementation possesses several advantages over the intramuscular approach, including fewer side effects, less pain and better compliance besides its lower serum level but increased progesterone level in the uterine endometria, thus leading to favorable effects on pregnancy rate^26,27^. However, no dosing method for transvaginal application of progesterone has been established currently and more studies are necessary for this.

It has been reported that progesterone supplementation can reduce the premature birth rate under certain conditions including artificial reproductive technologies^28^. Premature birth rates accounted reportedly for 5%–7% of newborns in developed countries^29^, and in the study by Enatsu *et al*.^2^, the premature birth rates accounted for 7.9% in the 1200 mg daily group and 8.7% in the 900 mg daily group of transvaginal progesterone supplementation. Progesterone also has certain effects on infant growth by ameliorating the mother’s appetite at pregnancy^2^, and Enatsu *et al*. had also reported normal birthweights at every gestational age on the chart of healthy Japanese birthweight^30^. Although there have been some reports reporting that there is no significant difference in the incidence of clinical pregnancy, continuous pregnancy, ectopic pregnancy, abortion and live birth between transvaginal and intramuscular progesterone supplementation^7,12,17,31^, our study with a large number of patients (3013) demonstrated that vaginal progesterone supplementation in sustained release had significantly (P < 0.05) greater implantation (37.0% vs 34.4%), delivery (45.1% vs. 41.0%) and live birth (45.0% vs. 40.8%) rate but significantly (P < 0.05) decreased abortion rate (15.3% vs. 19.4%) than the group of patients with intramuscular progesterone injection. In the FET cycle, the dose of progesterone vaginal sustained-release gel is usually 90 mg/d or 90 mg bid, and studies have shown that there is no difference in clinical effect between the two dosages^32,33^. So in our study, we used the sustained-release gel for vaginal progesterone supplementation with the dose of 90 mg/d. Sustained release of progesterone may help maintaining a certain concentration of progesterone in the uterus and is thus beneficial to good clinical outcomes. There are some studies with good outcomes for vaginal progesterone supplementation. After comparing vaginal gel supplementation and intramuscular injection of progesterone for external fertilization and transfer of embryo with GnRH-a protocol, Chi *et al*.^34^ found that the rate of implantation, clinical intrauterine pregnancy or live birth was significantly greater in the vaginal gel progesterone supplementation group than the intramuscular injection group. Silverberg *et al*.^21^ had investigated effects of vaginal progesterone gel in comparison with intramuscular injection of progesterone for support at the luteal phase in external fertilization and found that significantly increased pregnancy and delivery rates have been achieved in women receiving vaginal progesterone gel compared with those receiving intramuscular injection of progesterone. No significant differences were found in the incidences of biochemical or ectopic pregnancy and miscarriage between these two groups^21^. Ho *et al*.^35^ also found that vaginal gel supplementation of progesterone can significantly increase the implantation and pregnancy rate. However, other authors had found that there was no significant difference in the clinical outcomes including clinical pregnancy, implantation, live birth, ectopic pregnancy and abortion rates^7,12,17,31,36^. Because of these differences in the clinical outcomes between the two routs of progesterone supplementation, further studies are needed to confirm the effects of vaginal gel progesterone supplementation.

Some limitations may exist in this study including the retrospective nature, single center study, and single ethnicity of Chinese only. These conditions limit the results to be extended to other population or medical centers. Further prospective studies with multiple centers and people involved are needed to confirm the results of this study.

In conclusion, vaginal gel progesterone supplementation has good effects on frozen-thawed embryo transfer and can significantly increase the rate of implantation, delivery and live birth but decrease the abortion rate compared with intramuscular progesterone injection.

## References

[1] Trounson A, Mohr L (1983). Human pregnancy following cryopreservation, thawing and transfer of an eight-cell embryo. Nature.

[2] Enatsu Y (2018). Effectiveness of high-dose transvaginal progesterone supplementation for women who are undergoing a frozen-thawed embryo transfer. Reprod Med Biol.

[3] Schwartz E (2019). Luteal phase progesterone supplementation following induced natural cycle frozen embryo transfer: A retrospective cohort study. J Gynecol Obstet Hum Reprod.

[4] Seikkula J (2016). Effect of mid-luteal phase GnRH agonist on frozen-thawed embryo transfers during natural menstrual cycles: a randomised clinical pilot study. Gynecol Endocrinol.

[5] Shiotani M (2017). Is human chorionic gonadotropin supplementation beneficial for frozen and thawed embryo transfer in estrogen/progesterone replacement cycles?: A randomized clinical trial. Reprod Med Biol.

[6] Thomsen L H, Kesmodel U S, Erb K, Bungum L, Pedersen D, Hauge B, Elbæk H O, Povlsen B B, Andersen C Y, Humaidan P (2018). The impact of luteal serum progesterone levels on live birth rates—a prospective study of 602 IVF/ICSI cycles. Human Reproduction.

[7] Wang Y (2015). Crinone Gel for Luteal Phase Support in Frozen-Thawed Embryo Transfer Cycles: A Prospective Randomized Clinical Trial in the Chinese Population. PLoS One.

[8] Zarei A (2017). Comparison of four protocols for luteal phase support in frozen-thawed Embryo transfer cycles: a randomized clinical trial. Arch Gynecol Obstet.

[9] Ashrafi M, Jahangiri N, Hassani F, Akhoond MR, Madani T (2011). The factors affecting the outcome of frozen-thawed embryo transfer cycle. Taiwan J Obstet Gynecol.

[10] Lan VT, Tuan PH, Canh LT, Tuong HM, Howles CM (2008). Progesterone supplementation during cryopreserved embryo transfer cycles: efficacy and convenience of two vaginal formulations. Reprod Biomed Online.

[11] Nahoul K, Dehennin L, Jondet M, Roger M (1993). Profiles of plasma estrogens, progesterone and their metabolites after oral or vaginal administration of estradiol or progesterone. Maturitas.

[12] Tavaniotou A, Smitz J, Bourgain C, Devroey P (2000). Comparison between different routes of progesterone administration as luteal phase support in infertility treatments. Hum Reprod Update.

[13] Cercas R, Villas C, Pons I, Brana C, Fernandez-Shaw S (2012). Vitrification can modify embryo cleavage stage after warming. Should we change endometrial preparation?. J Assist Reprod Genet.

[14] Gordon JD (2013). Utilization and success rates of unstimulated *in vitro* fertilization in the United States: an analysis of the Society for Assisted Reproductive Technology database. Fertil Steril.

[15] Lee VC, Li RH, Ng EH, Yeung WS, Ho PC (2013). Luteal phase support does not improve the clinical pregnancy rate of natural cycle frozen-thawed embryo transfer: a retrospective analysis. Eur J Obstet Gynecol Reprod Biol.

[16] Nawroth F, Ludwig M (2005). What is the ‘ideal’ duration of progesterone supplementation before the transfer of cryopreserved-thawed embryos in estrogen/progesterone replacement protocols?. Hum Reprod.

[17] Shapiro BS, Daneshmand ST, Garner FC, Aguirre M, Ross R (2008). Contrasting patterns in *in vitro* fertilization pregnancy rates among fresh autologous, fresh oocyte donor, and cryopreserved cycles with the use of day 5 or day 6 blastocysts may reflect differences in embryo-endometrium synchrony. Fertil Steril.

[18] Veleva Z, Orava M, Nuojua-Huttunen S, Tapanainen JS, Martikainen H (2013). Factors affecting the outcome of frozen-thawed embryo transfer. Hum Reprod.

[19] Song Jian CF (2010). Deviation of Fertility Willingness and Behavior of Urban Youth and Its Influencing Factors: A Survey from Four Cities. Population Science of China.

[20] Vaisbuch E, Leong M, Shoham Z (2012). Progesterone support in IVF: is evidence-based medicine translated to clinical practice? A worldwide web-based survey. Reprod Biomed Online.

[21] Silverberg KM, Vaughn TC, Hansard LJ, Burger NZ, Minter T (2012). Vaginal (Crinone 8%) gel vs. intramuscular progesterone in oil for luteal phase support in *in vitro* fertilization: a large prospective trial. Fertil Steril.

[22] Eftekhar M, Rahsepar M, Rahmani E (2013). Effect of progesterone supplementation on natural frozen-thawed embryo transfer cycles: a randomized controlled trial. Int J Fertil Steril.

[23] Lightman A, Kol S, Itskovitz-Eldor J (1999). A prospective randomized study comparing intramuscular with intravaginal natural progesterone in programmed thaw cycles. Hum Reprod.

[24] Mitwally MF, Diamond MP, Abuzeid M (2010). Vaginal micronized progesterone versus intramuscular progesterone for luteal support in women undergoing *in vitro* fertilization-embryo transfer. Fertil Steril.

[25] Vaisbuch E, de Ziegler D, Leong M, Weissman A, Shoham Z (2014). Luteal-phase support in assisted reproduction treatment: real-life practices reported worldwide by an updated website-based survey. Reprod Biomed Online.

[26] Cicinelli E (2000). Direct transport of progesterone from vagina to uterus. Obstet Gynecol.

[27] Cicinelli E (2000). Mechanisms of uterine specificity of vaginal progesterone. Hum Reprod.

[28] Khandelwal M (2012). Vaginal progesterone in risk reduction of preterm birth in women with short cervix in the midtrimester of pregnancy. Int J Womens Health.

[29] Lawn JE (2006). 1 year after The Lancet Neonatal Survival Series–was the call for action heard?. Lancet.

[30] Itabashi K, Miura F, Uehara R, Nakamura Y (2014). New Japanese neonatal anthropometric charts for gestational age at birth. Pediatr Int.

[31] Zhu X, Ye H, Fu Y (2017). Comparison of neonatal outcomes following progesterone use during ovarian stimulation with frozen-thawed embryo transfer. Sci Rep.

[32] Alsbjerg B (2013). Increasing vaginal progesterone gel supplementation after frozen-thawed embryo transfer significantly increases the delivery rate. Reprod Biomed Online.

[33] Dal Prato L (2008). Vaginal gel versus intramuscular progesterone for luteal phase supplementation: a prospective randomized trial. Reprod Biomed Online.

[34] Chi HB (2018). Comparison of Vaginal Gel and Intramuscular Progesterone for *In vitro* Fertilization and Embryo Transfer with Gonadotropin-Releasing Hormone Antagonist Protocol. Chin Med J (Engl).

[35] Ho CH, Chen SU, Peng FS, Chang CY, Yang YS (2008). Luteal support for IVF/ICSI cycles with Crinone 8% (90 mg) twice daily results in higher pregnancy rates than with intramuscular progesterone. J Chin Med Assoc.

[36] Leonard PH (2015). Progesterone support for frozen embryo transfer: intramuscular versus vaginal suppository demonstrates no difference in a cohort. J Reprod Med.

